# G protein-coupled and ATP-sensitive inwardly rectifying potassium ion channels are essential for HIV entry

**DOI:** 10.1038/s41598-019-40968-x

**Published:** 2019-03-11

**Authors:** Ravi C. Dubey, Nawneet Mishra, Ritu Gaur

**Affiliations:** Faculty of Life Sciences and Biotechnology, South Asian University, New Delhi, 110021 India

## Abstract

The high genetic diversity of Human Immunodeficiency virus (HIV), has hindered the development of effective vaccines or antiviral drugs against it. Hence, there is a continuous need for identification of new antiviral targets. HIV exploits specific host proteins also known as HIV-dependency factors during its replication inside the cell. Potassium channels play a crucial role in the life cycle of several viruses by modulating ion homeostasis, cell signaling, cell cycle, and cell death. In this study, using pharmacological tools, we have identified that HIV utilizes distinct cellular potassium channels at various steps in its life cycle. Members of inwardly rectifying potassium (K_ir_) channel family, G protein-coupled (GIRK), and ATP-sensitive (K_ATP_) are involved in HIV entry. Blocking these channels using specific inhibitors reduces HIV entry. Another member, K_ir_ 1.1 plays a role post entry as inhibiting this channel inhibits virus production and release. These inhibitors are not toxic to the cells at the concentration used in the study. We have further identified the possible mechanism through which these potassium channels regulate HIV entry by using a slow-response potential-sensitive probe DIBAC4(3) and have observed that blocking these potassium channels inhibits membrane depolarization which then inhibits HIV entry and virus release as well. These results demonstrate for the first time, the important role of K_ir_ channel members in HIV-1 infection and suggest that these K^+^ channels could serve as a safe therapeutic target for treatment of HIV/AIDS.

## Introduction

Nearly 25, FDA approved drugs are available for the treatment of HIV/AIDS, but there is currently no cure for this disease^1–3^. HIV, being an RNA virus is prone to mutations during reverse transcription leading to diversity in its genome. In addition to the ongoing issues of drug tolerability and long-term adverse effects, treatment of drug-resistant strains has become a major problem that has limited options for many patients. Protease (PR) and reverse transcriptase (RT) inhibitors represent the backbone of the combination antiretroviral therapy^4,5^. However, during 15 years of widespread clinical applications, mutations that confer resistance to these drugs have accumulated. Hence, identification of new antiviral target continues to be a high priority for development of HIV therapeutics.

Genome-wide siRNA screen and protein-protein interaction studies have identified several cellular host factors required by HIV to perform different functions that are crucial for its replication^6–10^. In order to counteract HIV replication, it is important to target these host proteins as they are less prone to mutations compared to viral proteins. Efforts are being made to block HIV-1 entry by targeting cellular receptors/coreceptors^11,12^ and counteracting host antiviral response by modulating the interaction of viral proteins with host restriction factors^13^. Host ion channels (K^+^ and Cl^−^) are an emerging class of host factors that play an essential role in regulating ion homeostasis across membranes and are involved in several cellular processes including cell cycle, cell signaling, and cellular gene expression. The K^+^ channels are broadly classified in two groups: voltage-gated and ligand-gated which are further classified in 4 subfamilies: voltage-gated K^+^ channels (K_V_), inwardly rectifying K^+^ channels (K_ir_), two-pore K^+^ channels (K_2p_) and calcium-activated K^+^ channels (B_K_)^14^. These channels have been implicated to play a vital role during virus infection. Enveloped viruses such as Semliki forest virus and Human rhinovirus type 2 modulate membrane potential for their entry and release from the host cell by modulating host ion channels during their life cycle^15–17^. Certain viruses encode proteins called viroporins with ion channel properties whose function is essential for their life cycle making them ideal drug targets^18,19^. The HIV viral protein Vpu displays K^+^ channel activity to enhance virus release^20,21^. Additionally, the p13 protein encoded by HTLV-1 targets mitochondrial membrane potential that results in increased production of reactive oxygen species (ROS) by mitochondria^22,23^. The 6k protein encoded by Ross River virus forms a cation-selective ion channel which plays a role in its release^24,25^. Other viral proteins which display K^+^ channel activity to regulate virus infection include the 6 K protein of Sindbis and Semliki forest virus^26^ and Dengue virus C terminal peptide^27^. Modulation of potassium channels inhibits entry of Ebola virus^28^ and replication of Bunyavirus^29^. The potassium channels are also utilized by viruses to control cell death pathways. For example, the Hepatitis C virus non-structural protein NS5A modulates the function of Kv2.1, a voltage-gated K^+^ channel^27^ and regulates cell apoptosis. HIV-1 protein Nef alters the intracellular K^+^ ion concentration^30^ by targeting large-conductance Ca^2+^-dependent K^+^ channels (BK_Ca_)^31^ whereas viral Env protein, gp120 inhibits the voltage-gated K^+^ channel (BEC1) activity resulting in decreased virus release^32^. HIV gp120 induces hippocampal neuronal apoptosis by enhancement of Kv channel functions through p38 MAPK phosphorylation in HIV associated neurocognitive disorder^33^.

In this study, we have systematically analyzed the role of K^+^ channels in the entry, replication, and release of HIV-1 virus using pharmacological tools. We have observed that members of the K_ir_, GIRK and K_ATP_ are involved in HIV entry whereas K_ir_ 1.1 plays a role in the release of HIV. We have also elucidated the mechanism of action of these channels and observed that blocking these channels inhibited membrane depolarization which reduced HIV entry. We propose that potassium channels may be further explored as new, pharmacologically safe HIV therapeutics.

## Results

### Potassium Channels in HIV Production

**Figure 1:**
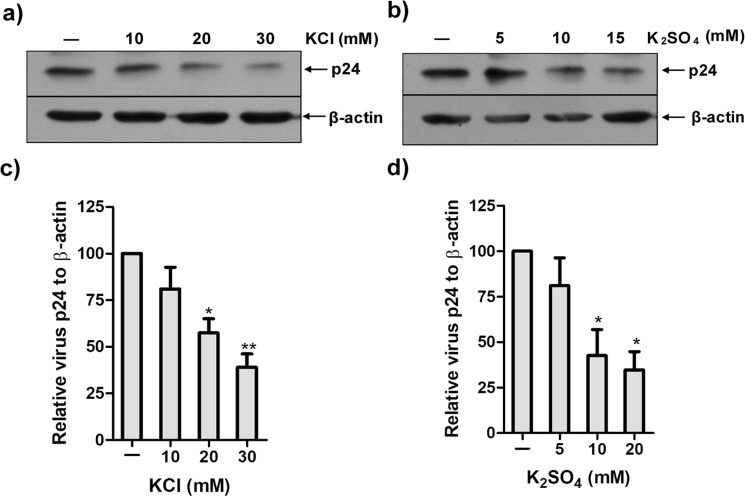
Increased K^+^ ion concentration in media inhibits HIV production. (**a**,**b**) MT4 cells were infected with HIV (NL4-3) for 2 hrs and then incubated with indicated concentration of K^+^ salt for 48 hrs. The relative virus production was determined in cell lysate by western blotting. **(c,d)** Graphs represent the p24 band intensity normalised by β-actin. Values represent relative change in p24 level ± SEM derived from three independent experiments (* denotes p < 0.05; ** denotes p < 0.01, ANOVA).

**Figure 2:**
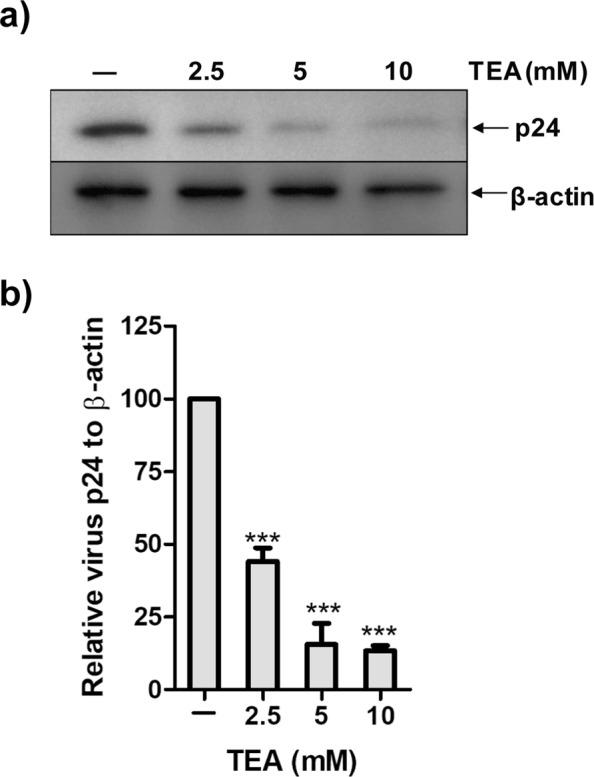
Increased TEA concentration in media inhibits HIV production. (**a**) MT4 cells were infected with HIV (NL4-3) for 2 hrs and then incubated with indicated concentration of TEA for 48 hrs. The relative virus production was determined in cell lysate by western blotting. (**b**) Graphs represent the p24 band intensity normalised by β-actin. Values represent relative change in p24 level ± SEM derived from three independent experiments (*** denotes p < 0.001, ANOVA).

HIV-1 induces an increase in intracellular K^+^ concentration for efficient viral replication^34^. To determine this, we measured virus production in the presence of two potassium salts: KCl and K_2_SO_4_. MT4 cells were infected with NL4-3 (HIV-1) and incubated in the presence of increasing concentration of KCl or K_2_SO_4_ for 48 hrs. Virus production was measured by detecting expression of the HIV-1 capsid protein, p24 by western blotting. An increase in salt concentration inhibited the virus production in a dose-dependent manner (Fig. 1a–d). Nearly 65–70% reduction in p24 was observed at 30 mM concentration of KCl or 20 mM K_2_SO_4_ relative to β-actin. TEA, a broad-spectrum potassium channel blocker^35,36^, reduced virus production by 90% (Fig. 2a,b). These observations suggest that potassium channels may play a vital role in the life cycle of HIV.

### Potassium Channels in HIV Entry

There are three major steps in the virus life cycle: virus entry, replication, and release. We proposed to investigate the role of potassium channels in modulating these three significant steps of HIV life cycle using specific inhibitors as mentioned in Table 1. 4-Aminopyridine (4-AP) blocks K_V_ channel activity by delaying the burst time of the channel^37^. The activity of Kir 1.1 channels can be blocked by using BaCl_2._ Ba^+^ ions blocks cation movement by replacing the ion at the internal side of the central cavity of the channel^38,39^. BK channel activity can be inhibited by CoCl_2_. Cobalt inhibits the Ca^++^ current which reduces the K^+^ current through BK channels^38,39^. K_ATP_ and GIRK can be targeted by Glibenclamide and Ifenprodil respectively. Glibenclamide blocks the channel activity from either side of the membrane^40^ while Ifenprodil reversibly reduces the inward current by targeting GIRK from the extracellular side^40^.

**Table 1:** Different Potassium channel blockers used.

Compound	K^+^ channels blocked
TetraethyleneAmmonium (TEA)	Broad spectrum
4-Aminopyridine (4-AP)	Kv channel
BaCl_2_	Kir 1.1 channel
CoCl_2_	BK channel
Glibenclamide (Glib)	K_ATP_ channel
Ifenprodil (Ifen)	GIRK channel

**Figure 3:**
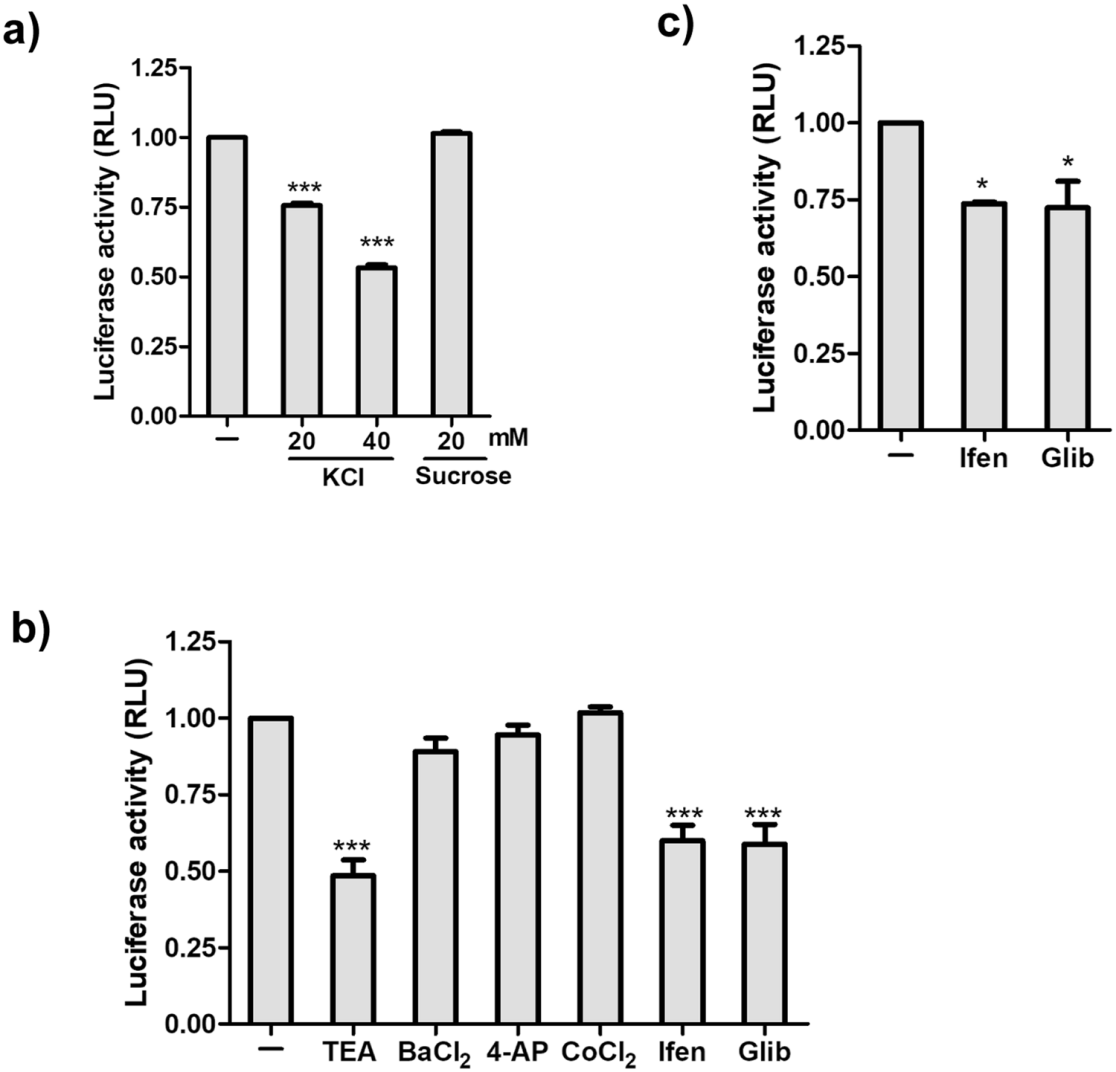
Effect of K^+^ channel inhibitors on HIV entry. (**a**,**b**) TZMbl cells were preincubated with increased concentration of KCl or K^+^ channel blockers (TEA 5 mM, BaCl_2_ 1 mM, 4-AP 1 mM, CoCl_2_ 50 uM, Ifen 200 uM, Glib 200 uM) for 1 hour followed by HIV infection. Luciferase expression was measured in the cell lysates 48 hrs post infection. (**c**) TZMbl cells were infected with HIV (NL4-3) in the presence of compounds without pre-treatment. Graph represents the measured luminescence in the cells. Values represent relative change ± SEM derived from 3 independent experiments (* denotes p < 0.05; *** denotes p < 0.001, ANOVA).

We first examined the effect of family-specific K^+^ channel blockers on HIV entry in the host cells. Each blocker was used at a concentration which was not toxic to the cells (data not shown). For our experiments, we have used the most conventional system, the TZMbl assay which is based on the expression of the luciferase gene under the HIV-1 long terminal repeat (HIV-LTR) promoter. These cells have been widely used to study HIV entry and infection cycle. The TZMbl cells were pre-incubated with the indicated concentration of each blocker for 1 hr followed by HIV infection in the presence of the blockers for additional 2 hrs. The cells were washed post infection and further incubated for 48 hrs in the absence of blockers. Virus production was estimated by measuring the luciferase gene expression. Increased K^+^ ion concentration in media reduced virus entry by nearly 50%. The decrease in virus entry was not due to changes in osmolarity of the media as the addition of sucrose did not produce the same effect (Fig. 3a). TEA, a broad-spectrum K^+^ channel blocker inhibited HIV entry by 50%. Interestingly, among all K^+^ family blockers, only Ifenprodil, and glibenclamide reduced HIV entry by 50% which was reflected by decreased relative luminescence units (RLUs) (Fig. 3b). A similar decrease was observed even without pre-incubation with these compounds (Fig. 3c). No effect on virus entry was observed with other K^+^ channel blockers, 4-AP, BaCl_2_, and CoCl_2_, indicating that these families are not involved in HIV entry. Taken together, these results suggest that K^+^ channel subfamilies: K_ATP_ and GIRK actively participate in HIV entry.

### Potassium Channels in HIV Replication

**Figure 4:**
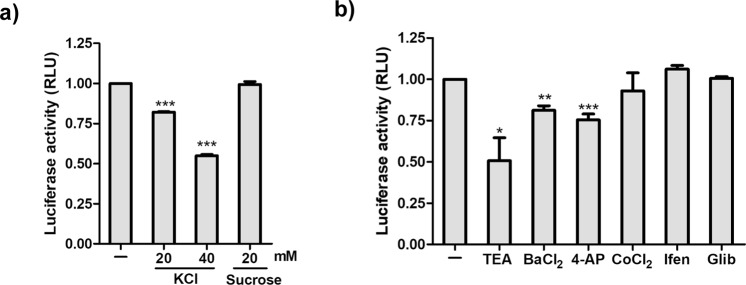
Effect of K^+^ channel blockers on HIV replication. (**a**) TZMbl cells were infected with HIV (NL4-3) followed by incubation for 48 hrs with KCl (20 and 40 mM) and sucrose (20 mM). (**b**) TZMbl cells were infected with HIV (NL4-3) followed by incubation with K^+^ channel blockers (TEA 5 mM, BaCl_2_ 1 mM, 4-AP 1 mM, CoCl_2_ 50 uM, Ifen 50 uM, Glib 50 uM) for 48 hrs. Graphs represent the luminescence detected in the infected cells. Values represent relative change ± SEM derived from 3 independent experiments (* denotes p < 0.05; ** denotes p < 0.01, *** denotes p < 0.001, ANOVA).

Next, we wanted to determine if potassium channels play a role at other stages of HIV life cycle post-entry. TZMbl cells were infected with the virus in the absence of various blockers. The cells were washed post infection and incubated in the presence of KCl, sucrose or specific blockers for 48 hrs. We observed a dose-dependent reduction in cell-associated luminescence after addition of KCl which confirmed that K^+^ ions play a role post–entry of the virus. This effect is not due to changes in osmolarity as the addition of sucrose did not affect cell-associated luminescence (Fig. 4a). We further observed that addition of inhibitors 4-AP (K_v_ channel), and BaCl_2_ (K_ir_ 1.1 channel) reduced virus production by 25% as evident from the decrease in cell-associated luminescence (Fig. 4b). This could be due to a reduction in Tat production due to the inhibition of virus replication inside the cells. Interestingly, other inhibitors such as CoCl_2_ (B_K_ channels), Ifenprodil (GIRK) and glibenclamide (K_ATP_) did not inhibit virus replication when added post virus entry (Fig. 4b). Together, these results suggest that different K^+^ channel families are involved at various stages of the HIV life cycle.

### Potassium Channels in HIV Release

**Figure 5:**
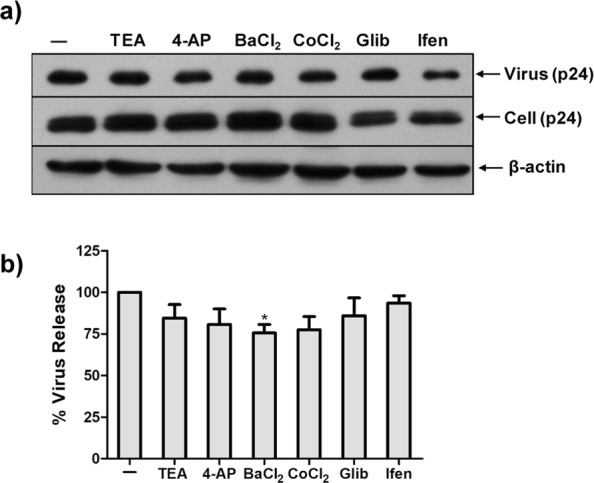
Effect of K^+^ channel blockers on HIV release. (**a**) HEK293T cells were transfected with pNL4-3 and incubated with indicated concentration of K^+^ channel blockers for 24 hrs. The relative virus production was determined in supernatant and cell lysate by western blotting. (**b**) Graphs represent the % virus release calculated using the formula % p24 release = [p24 in supernatant/(p24 in supernatant + p24 in cell)] × 100. ±SEM derived from three independent infections (* denotes p < 0.05, ANOVA).

Next, we were interested in determining if K^+^ channels participate in HIV release. We transfected HEK293T cells with pNL4-3 DNA and incubated the cells post-transfection with various inhibitors for 24 hrs. Virus release was measured by detecting the expression of p24 in the culture supernatant by immunoblotting (Fig. 5a,b). We observed a 25% decrease in virus release in the presence of BaCl_2_ indicating that Kir 1.1 family of potassium channels might be involved in the virus release. We observed a slight increase in cellular p24 with BaCl_2_ which could either be due to a direct effect of BaCl_2_ on increasing virus production or could be due to an accumulation of p24 in the cell as a result of a block in virus release. A slight decrease in virus release was also observed in the presence of CoCl_2_ and 4-AP, but it was statistically insignificant. It is important to note that although TEA is a broad spectrum K^+^ channel blocker, it is not highly sensitive for Inwardly rectifying K^+^ channels as compared to BaCl_2_ which is highly specific^38,39,41^ Hence, we did not observe a similar effect on virus release with TEA.

### Membrane potential and HIV entry

**Figure 6:**
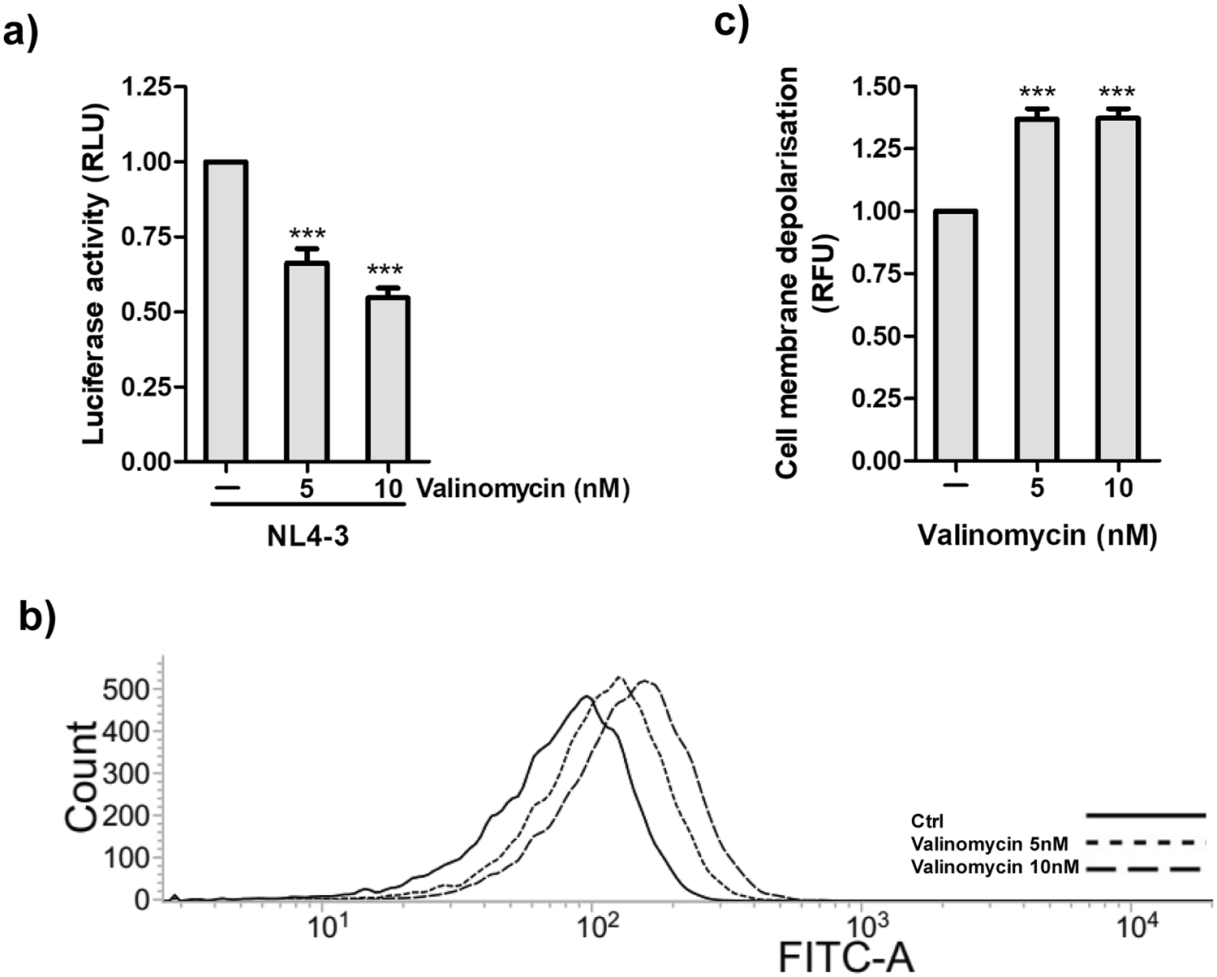
Valinomycin represses HIV entry and depolarises the cell membrane. (**a**) TZMbl cells were infected with HIV in the presence or absence of valinomycin for 2 hrs. Graphs represent the luminescence detected in the infected cells. (**b**) TZMbl cells were treated with Valinomycin for 2 hrs followed by incubation with DIBAC4(3) (250 nM) for 30 min at room temperature (**c**) Bar Graph represent relative mean fluorescence in the cells. Values represent relative change ± SEM derived from 3 independent experiments (*** denotes p < 0.001, ANOVA).

We further investigated if membrane potential plays any role in modulating HIV entry, using slow-response potential-sensitive probe DIBAC4(3). This probe enters depolarized cells where it binds to intracellular proteins or membrane, and exhibits enhanced fluorescence on binding^40^. TZMbl cells were treated with valinomycin (a K^+^ ionophore) before HIV infection. Valinomycin at 10 nM concentration impeded virus entry by nearly 50% (Fig. 6a) and depolarised the membrane as evident from increased fluorescence obtained with DIBAC4(3) (Fig. 6b,c). These results suggested that depolarization of membrane inhibits HIV entry.

### Role of Potassium channel blockers in modulating membrane potential

**Figure 7:**
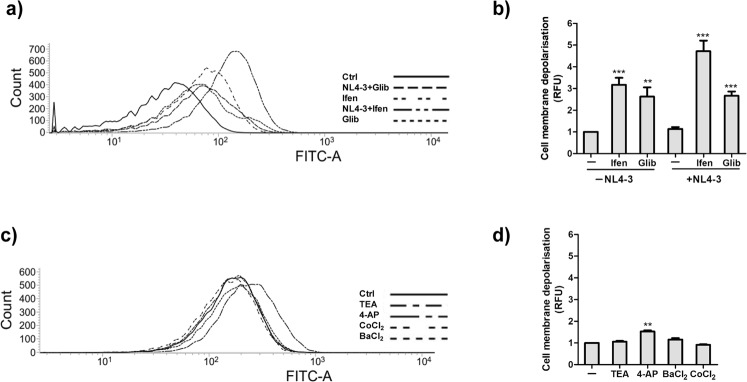
Effect of K^+^ channel blockers on membrane potential. (**a**–**c**) HIV infected/uninfected TZMbl cells were treated with indicated concentration of the compounds for 2 hrs. The cells were incubated with DIBAC4(3) (250 nM) for 30 min at room temperature followed by FACS analysis. (**b**–**d**) Bar Graphs represent relative mean fluorescence in the cells. Values represent relative change ± SEM derived from 3 independent experiments (** denotes p < 0.01; *** denotes p < 0.001, ANOVA).

Next, we hypothesized that potassium channel blockers might also reduce virus entry by increased depolarization of cells. To test this hypothesis, we have measured membrane depolarization in the presence of various potassium channel blockers. We have observed that the inhibitors Ifenprodil and Glibenclamide depolarised the cells by reducing K^+^ efflux as evident from the increased fluorescence obtained with DIBAC4(3) (Fig. 7a,b) in the absence of HIV-1. Presence of Ifenprodil during HIV infection further enhanced fluorescence. These results suggested that K_ATP_ and GIRK channel family may play a role in hyperpolarization of cells which is essential for virus entry. Similarly, presence of 4-AP also slightly increased membrane depolarisation suggesting its role during virus replication inside the cells (Fig. 7c,d). Presence of TEA and other K^+^ family blockers had no effect on membrane potential. (Fig. 7c,d).

## Discussion

Potassium channels play a key role in maintaining K^+^ ion homeostasis and membrane potential and are involved in many cellular processes. These ion channels serve as an ideal therapeutic target in several diseases^14^. It has been previously reported that HIV virus production, in RH9 cells infected with HIV-1 strain LAI, decreases with increasing KCl concentration in media suggesting that changes in extracellular potassium ions modulate HIV gene expression^34^, but the specific role of different potassium channel families during HIV life cycle is not known. In the current study, we have analyzed the role of specific K^+^ channel families at different steps in the HIV life cycle using available pharmacological inhibitors.

We have observed that elevated concentration of both the K^+^ salts KCl and K_2_SO_4_ reduced HIV virus production (Fig. 1a–d). Addition of broad-spectrum K^+^ channel blocker, TEA also diminished virus production suggesting that the effect of K^+^ ions on virus production is specific and not due to changes in osmolarity which may also decrease cellular protein synthesis^42^. Hence, these results imply that HIV may utilize multiple K^+^ channels during its life cycle (Fig. 2a,b).

The GIRK channels are present on the plasma membrane, and their opening is mediated by G protein-coupled receptors (GPCR)^43^. The GPCRs associated with GIRK are not involved in signal transduction pathways but directly activate these ion channels using effector proteins or the G protein subunits themselves. Several GPCRs are members of the chemokine receptor subfamily. The HIV coreceptor CCR5 is an example of GPCR which is involved in HIV entry^44^. Our study demonstrates that GIRK subfamily of potassium channels are involved in virus entry. Blocking these channels with Ifenprodil impeded HIV entry by 50% (Fig. 3b).

The K_ATP_ channels are widely distributed and present in several tissues. Their activity is regulated by the intracellular pool of Adenine nucleotides linking cellular metabolism with membrane excitability. In the early steps of HIV infection, viral protein p2 increases the cellular ATP pool which facilitates reverse transcription^45^. Our results highlight that these channels play a significant role in HIV entry. Blocking the K_ATP_ channel with glibenclamide also inhibits viral entry (Fig. 3b).

Changes in membrane potential regulate cell viability and cell cycle. Role of membrane potential in the fusion and entry of semliki forest virus has been demonstrated^15^. HIV Nef protein alters the membrane potential of the HIV infected cell. Overexpression of Nef hampers the activity of Ca^+^^2^ dependent K^+^ channels^45^. Another HIV protein, Vpu, interacts with a cellular weak inward K^+^ rectifier TASK1 and plays a crucial role in the enhancement of virus release from the host cell by causing membrane potential depolarization^46^. HIV envelop protein, gp120, induces cell death in neuronal cells by targeting K^+^ channels^46–48^.

To understand the role of membrane potential in HIV entry, we used a fluorescent membrane potential probe DIBAC 4(3) and observed that a K^+^ ionophore Valinomycin inhibited HIV entry by increasing membrane depolarization. This suggests that membrane hyperpolarisation is essential for HIV entry. We observed a similar increase in membrane depolarization in the presence of both Ifenprodil and Glibenclamide implying that these compounds may also block virus entry by depolarization of the membrane (Fig. 7a,b). This is the first report of a possible mechanism of action of Ifenprodil. Glibenclamide is already being used in the treatment of type 2 diabetes and works by causing membrane depolarization in pancreatic cells leading to release of insulin^47–49^. However, other mechanisms of action of these compounds through cell signaling or any other pathway needs to be further investigated.

The K_V_ channels play a role in the progression of HIV associated dementia (HAD)^33,50^. Blocking the Kv channels with 4-AP inhibited intracellular virus production (Fig. 4b).

It is interesting to note that a non-specific K^+^ channel blocker TEA, inhibited HIV production without altering membrane potential inferring that there might be other mechanism involved in this process also (Figs 3b and 7c,d).

Together, our study highlights the important role played by K_ir_ channel families at different steps in the HIV life cycle and suggest these K_ir_ channels may be pursued as new therapeutic targets for HIV infection.

## Conclusion

To address the challenge of drug resistance, it is necessary to unravel new targets that could be developed as alternate HIV therapeutics. We have demonstrated that K_ir_ potassium channels play a vital role at different steps of HIV life cycle and could serve as novel targets for the better management of HIV/AIDS.

## Methods

### Cells, Virus, and Drugs

MT4 cells were propagated in RPMI1640 supplemented with 10% (v/v) fetal bovine serum (FBS). TZM-bl and HEK-293T cells were propagated in Dulbecco’s modified Eagle’s medium (DMEM) containing 10% (v/v) FBS. HIV-1 molecular clone NL4-3 (a kind gift from Dr. Eric O Freed, NCI, NIH, USA) was used to produce infectious virus by transfecting HEK293T cells. For transfection, the cells were seeded in 6 well tissue culture plate at 0.3 × 10^6^ cells/well. At about 70% confluency, the cells were transfected with 1 μg DNA using Lipofectamine 2000 (Invitrogen, USA). The supernatant was harvested and filtered with 0.45 μm membrane filter. Viral p24 concentration was measured by using a p24 antigen capture ELISA kit (ABL, USA). Potassium chloride (cat: P9541) and cobalt chloride (cat: 232696) was purchased from Sigma-Aldrich,USA. Tetraethylammonium chloride (cat: T0095) and Glibenclamide (cat: G0382) was purchased from TCI chemicals (Japan). Barium chloride dihydrate (cat: 101719) and Ifenprodil (cat: 17201) was purchased from Cayman chemical company (USA) and Merck Life sciences (USA) respectively. 4-Aminopyridine (cat: J61470) was purchased from Alfa-Aesar (Britain). Potassium sulfate (cat: GRM1403) was purchased from HiMedia (India) Ifenprodil and Glibenclamide were dissolved in DMSO while others were dissolved in MQ water.

### Western Immunoblotting

MT4 cell were infected with NL4-3 for 48 hrs and lysed in radioimmunoprecipitation assay (RIPA) buffer (50 mM Tris-HCl pH 8.0, 150 mM sodium chloride, 1.0% NP-40, 0.5% sodium deoxycholate, 0.1% SDS) containing 1× protease inhibitor cocktail (Roche, Germany). The cell lysate was resolved by running on a 12% SDS-polyacrylamide gel. The proteins were transferred to polyvinylidene difluoride (PVDF, Millipore, USA) membrane by electrophoretic transfer method and probed with human HIV serum (NIH AIDS Reagent Program cat: 3957) followed by labeling with anti-human HRP conjugated secondary antibodies (GE Healthcare, UK). The western blots were visualized with ECL (Pierce, USA) system followed by exposure to X-ray films. Bands on the X-ray films were analyzed with ImageJ software (http://imagej.nih.gov/ij/).

### Luciferase Assay

TZMbl cells were seeded in 24 well tissue culture plate at 0.05 × 10^6^ cells/well and infected with HIV in the presence of the compounds. After 2 hrs of incubation, cells were washed twice with PBS and further incubated at 37 °C for 48 hrs. The cells were lysed post-infection with steady Glo-lysis buffer (Promega, USA) and luminescence was measured in a microplate reader as per manufacturer’s instructions.

### Virus Release Assay

HEK293T cells were seeded at 0.1 × 10^6^ cells/well in a 24-well plate and transfected with 1 μg of DNA/well using Lipofectamine 2000. After 24 hrs, the viral supernatant and cell lysate were harvested and separated on 12% SDS-PAGE gel and proteins were transferred to PVDF membrane. The membrane was probed with HIV-IgG obtained from the NIH AIDS Reagent Program (cat: 3957) followed by HRP-conjugated human secondary antibody (GE Healthcare, UK). The western blots were visualized with ECL (Pierce, USA) system followed by exposure to X-ray films. The band intensity was analysed with ImageJ software (http://imagej.nih.gov/ij/). The p24 in the cell was normalised for β actin. Percent release of the virus was calculated using following formula: % p24 release = [p24 in supernatant/(p24 in supernatant + p24 in cell)] × 100.

### Membrane Potential

The dye DIBAC4(3) (Invitrogen, Inc, USA) was used to measure changes in the membrane potential of the cells by flow cytometry as described previously^51,52^. Briefly, the cells were infected with the virus or treated with compounds for 2 hrs followed by addition of DIBAC4(3) (250 nM) for 30 min at 37 °C. The fluorescence was measured using FACS at 530 nm.

### Cytotoxicity assay

Toxicity of the used drugs on the cells was evaluated by using Cell Titre-Blue Cell Viability Assay kit (Promega, USA). Cells were seeded in 96 well tissue culture plate at 10,000 cells/well and treated with drugs at the used concentration in this study. The cell was treated with CellTitre-Blue reagent for 4 hrs at 37 °C, and the fluorescence was measured with BioTek microplate reader at 530/25_excitation_ and 590/35_emission_.

### Statistical Analysis

One-way ANOVA test was used to assess statistical differences between test and control samples cultured in the presence or absence of the compounds.
